# PRIAS: An Intelligent Analysis System for Pesticide Residue Detection Data and Its Application in Food Safety Supervision

**DOI:** 10.3390/foods11060780

**Published:** 2022-03-08

**Authors:** Yi Chen, Haifeng Dou, Qiaoying Chang, Chunlin Fan

**Affiliations:** 1 Beijing Key Laboratory of Big Data Technology for Food Safety, Beijing Technology and Business University, Beijing 100048, China; dhaifeng@yeah.net; 2 Chinese Academy of Inspection and Quarantine, Beijing 100176, China; c81618@163.com (Q.C.); caiqfcl@163.com (C.F.)

**Keywords:** pesticide residue, intelligent analysis system, statistical analysis, association rule, fusion processing

## Abstract

Pesticide residue is a prominent factor that leads to food safety problems. For this reason, many countries sample and detect pesticide residues in food every year, which generates a large amount of pesticide residue data. However, the way to deeply analyze and mine these data to quickly identify food safety risks is still an unresolved issue. In this study, we present an intelligent analysis system that supports the collection, processing, and analysis of detection data of pesticide residues. The system is first based on a number of databases such as maximum residue limit standards for the fusion of pesticide residue detection results; then, it applies a series of statistical methods to analyze pesticide residue data from multiple dimensions for quickly identifying potential risks; it uses the Apriori algorithm to mine the implicit association in the data to form pre-warning rules; finally, it applies Word document automatic generation technology to automatically generate pesticide residue analysis and pre-warning reports. The system was applied to analyze the pesticide residue detection results of 42 cities in mainland China from 2012 to 2015. Application results show that the system proposed in this study can greatly improve the depth, accuracy and efficiency of pesticide residue detection data analysis, and it can provide better decision support for food safety supervision.

## 1. Introduction

Pesticides, which are a class of agrochemicals for preventing and controlling pests and regulating plant growth, are widely used in the growth of edible agricultural products. However, the excessive use of pesticides will threaten human life and cause damage to human health [1,2,3]. For this reason, many countries have formulated monitoring programs for pesticide residues in food and regularly conduct random inspections on the pesticide residue content in locally marketed food/agricultural products to assess the levels of pesticide residues in foods and safety risk [4]. For example, the U.S. Food and Drug Administration (FDA) has been implementing the pesticide residue monitoring program since 1987 [5], sampling and monitoring pesticide residues in domestic and imported food every year and releasing monitoring reports and data. The European Food Safety Authority (EFSA) organizes its member states to monitor pesticide residues in food and publishes reports every year [6]. China’s State Administration for Market Regulation (SAMR) conducts random inspections on marketed foods and releases inspection results every month. SAMR has also established a Food Safety Inspection Result Query System [7]. Users can search inspection results since 2014, including information on qualified and unqualified foods. Excessive pesticide residues are one of the main reasons for unqualified foods. Pesticide residues are an important aspect of food safety supervision [8].

The emergence and development of detection techniques such as enzyme inhibition [9], immunoassay [10], spectroscopic detection [11,12,13], and mass spectrometric detection [14] have made the detection of pesticide residues in agricultural products more efficient and accurate, and the amount of detection data obtained is also increasing. These detection results are interwoven with information on agricultural products, pesticides, geographic regions, and maximum residue limit (MRL) standards, which forms a large number and complex relationship of pesticide residue datasets [15,16]. On the one hand, this situation provides a wealth of data resources for formulating pesticide residue control measures. On the other hand, it also poses great challenges to the collection, storage, analysis, and mining of pesticide residue data [17]. The emergence of food safety big data [18] has enabled data-driven risk analysis and pre-warning techniques to play an increasingly important role in food safety supervision [19,20,21]. 

Statistics, data mining, and other intelligent techniques have been applied to data analysis in the field of food safety, which has greatly improved the efficiency of food safety risk analysis, discovery, pre-warning, and tracing and provided new means for food safety detection and control. Statistical analysis methods are often used to discover distribution characteristics and outliers in food safety data. For example, Kuuliala et al. used multivariate statistical analysis to explore potential factors that lead to seafood spoilage [22]. Al-Shamary et al. analyzed pesticide residues in Qatari fruits and vegetables using the t-test method and found differences in pesticide residues in washed and unwashed samples [23]. Szarka et al. proposed a new statistical method that combines robust regression on ordered statistics with a maximum-likelihood estimator to quantify pesticide residue concentrations in the presence of heavily censored datasets, and they found that the median is a more robust measure of central tendency than the mean [24]. Data mining techniques are often applied to discover knowledge and uncover hidden rules and potential associations in food safety data. Muangprathub et al. used the Apriori algorithm to analyze crop growth monitoring data for mining the relationship among temperature, humidity, and soil moisture to optimize the future growth environment of crops [25]. Wang et al. used the association rule mining algorithm (Apriori) to mine association rules from inspection data of the food supply chain, and then, they generated warning rules to provide pre-warning of potential food safety risks [26]; Rong et al. used the Apriori algorithm to study cooking recipes to mine the correlation among ingredients, flavor, cooking time, cooking methods, and other information for helping people better match ingredients in cooking [27].

Statistical analysis methods and data mining techniques have played a good role in food safety data analysis. However, these methods are still independent of one another and have not been integrated into a system. A large number of manual operations are still required in the analysis process, such as MRL queries for various pesticides in various agricultural products, the determination of the contamination level of residues, multidimensional analysis of pesticide residue data, and editing analysis reports, which results in relatively low efficiency and accuracy of data analysis. The design and development of a set of collection, storage, analysis, and automatic generation of analytical reports of an intelligent analysis system are imperative. Information systems have been widely used in the food industry with good results due to their ability to process and manage data efficiently [28,29,30]. Integrating statistical analysis and data mining techniques into information systems to form an intelligent analysis system will greatly improve the efficiency of data processing, the quality of data management, and the utilization of data resources and provide better decision support for food safety supervision. According to the research, no public reports of similar work have been identified.

For these reasons, we designed an intelligent analysis system (PRIAS) for pesticide residue detection data to support the automatic collection, storage, analysis of pesticide residue data, and ultimate generation of pesticide residue detection data analysis reports. The contributions of this work are as follows.

(1) A data fusion method of pesticide residues based on the database is proposed. The method can achieve error checking and information supplementation of the original data, as well as determining the contamination level of residues. (2) On the basis of statistical methods, several pesticide residue statistical indicators are designed to help users analyze the distribution characteristics of pesticide residue contamination from multiple perspectives, such as sampling areas, agricultural products, pesticides, and discover high-risk pesticides, agricultural products, and geographical areas. (3) Association rule mining technology is applied to mine the internal association implied in the pesticide residue data for discovering potential food safety risks and pre-warning. (4) Word document generation technology is used, and a complete result analysis report is automatically generated according to the user’s analysis requirements. The report is presented in the form of text, data, tables, and statistical graphs, including the above-mentioned statistics and mining results, as well as the conclusions and pre-warning information obtained from them, which can provide decision support for food safety supervision. The pesticide residue detection data from mainland China from 2012–2015, as a case study, are applied to PRIAS for analysis, and the analysis results verify the feasibility of the methodology in this study.

## 2. Pesticide Methods

### 2.1. PRIAS Framework

**Figure 1:**
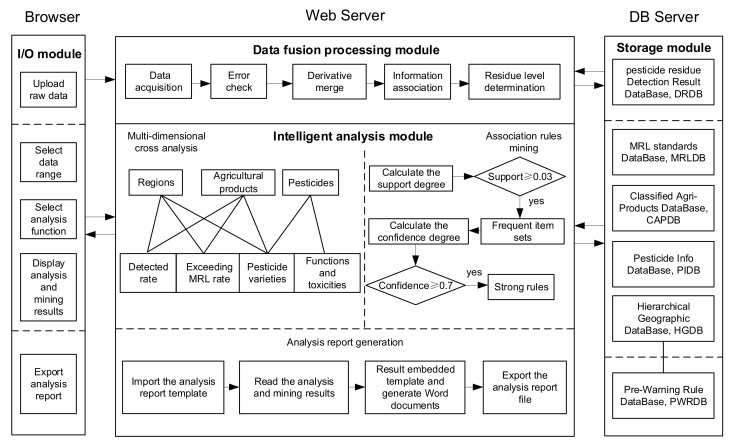
Framework of intelligent analysis system for Pesticide residue detection data.

PRIAS adopts Browser/Server mode and consists of an I/O module, storage module, data fusion processing module, and intelligent analysis module, as shown in Figure 1.

In the framework, the I/O module allows users to upload detection results, select the data ranges and analysis function, display the analysis and mining results in the form of figures and texts, and export the complete result analysis report document. The storage module is used to store detection results, relevant basic information, and pre-warning rules. The relevant basic information includes MRL standards, agricultural classification information, pesticide information, and hierarchical geographical information, which are used to assist in data processing and analysis. The data fusion processing module realizes error checking, derivative merging, and information association of the original data and determines the residue level. Thereafter, the fusion processed data are stored in the detection result database. The intelligent analysis module realizes multidimensional cross-analysis and association rule mining on the detection result data in the database to help users understand the characteristics of data distribution and mine the internal association between data. This module also generates a complete result analysis report automatically with the analysis results as a basis.

The advantage of the Browser/Server architecture adopted by PRIAS is that client programs need not be installed and maintained on the user side, which can be accessed by the user through a browser. Detection institutions (users) distributed in different regions regularly upload pesticide residue detection results to the Web Server through their own browsers, and the Web Server is based on the analysis method and the information in the database of the detection results for fusion processing and intelligent analysis, to obtain the analysis results and complete analysis report, returned to the user browser.

### 2.2. Database Design

The pesticide residue dataset mainly includes six types of information: pesticide residue detection results, pesticide basic information, classification information of agricultural products, administrative divisions of sampling areas, MRL standards, and pre-warning rules. They are described as follows.

**Figure 2:**
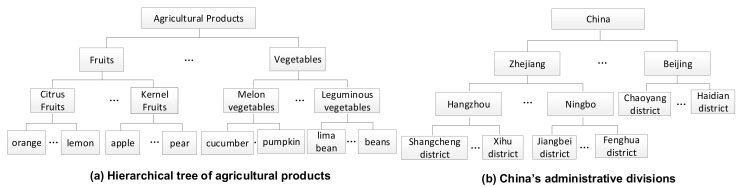
(**a**) Classification hierarchy of Chinese agricultural products and (**b**) geographical hierarchy of China.

(1) The detection results mainly include sampling time, sampling point, sample name, and the name and content of the pesticide detected.
(2) Pesticide information includes pesticide name, chemical composition, function, toxicity, and derivative. Among them, the chemical composition includes organochlorine, organophosphorus, carbamate, pyrethroid, organic nitrogen, or organic sulfur pesticide. The function includes insecticides, fungicides, herbicides, and plant growth regulators. Toxicity can be low, medium, high, or severe.
(3) The classification of agricultural products is organized in a hierarchical structure, as shown in Figure 2a, and includes primary, secondary, and tertiary categories. The primary classification can take the values of fruits, vegetables, and so on. The secondary classification can be citrus fruits, melon vegetables, and so on. The tertiary classification can take the values of orange, cucumber, and so on.
(4) Sampling points are usually supermarkets or farmers’ markets. The geographical areas (China) to which they belong are also organized in a hierarchy, including geographic regions (e.g., East China and North China), provinces level (e.g., Zhejiang and Beijing), cities (e.g., Hangzhou and Zhangjiakou), and counties (e.g., Shangcheng and Xihu), as shown in Figure 2b.
(5) The MRL standard specifies the maximum limit of each pesticide in specific agricultural products, which is the basis for determining the residue level. It mainly involves the names of pesticides, agricultural products, and limit values.
(6) The pre-warning rules are the results obtained after mining pesticide residue data through association rules, including rule’s antecedent items, rule’s subsequent items, support, and confidence. The item set includes information on agricultural products, sampling area, detected pesticides, the chemical composition of detected pesticides, the toxicity of detected pesticides, and function of detected pesticides.

The pesticide residue dataset containing the six types of information mentioned above is a multidimensional dataset with hierarchical, spatiotemporal, and interrelated characteristics. Considering the characteristics of making data structured, easy to relate and share, satisfying independence, and low redundancy, six databases are designed according to the data attributes and the linkage among them. They are pesticide residue Detection Result Database (DRDB), MRL Standard Database (MRLDB), Pesticide Info Database (PIDB), Classified Agricultural Product Database (CAPDB), Hierarchical Geographic Database (HGDB), and Pre-Warning Rule Database (PWRDB). Table 1 shows the main properties of the six databases. In this study, MRL is using GB2763-2014, which is issued by the Chinese government [31].

### 2.3. Data Fusion Processing

Raw detection result data usually have the following problems. (1) The format is not standardized. For example, the format of the sampling time exists in various formats, such as “2015/12/23,” “20151223,” and “2015-12-23,” which needs to be standardized. (2) The name is not standardized. Some agricultural products have different names in different regions. Thus, each agricultural product should have a unique standardized name. For example, “xihongshi” and “fanqie” represent the same agricultural product in Chinese and need to be standardized as “tomato.” (3) Incomplete information. For example, the name of agricultural products does not contain classification information, but detecting the residue value is necessary for comparison with MRL standards and supplementation, according to the classification of agricultural product standards. (4) Determining the pesticide residue level by comparing the detected pesticide residue content with MRL is also important. Therefore, the raw data are normalized by data fusion processing and data from multiple sources are fused into a complete dataset with better performance according to some rules to obtain a more accurate and comprehensive data description than a single source [32].

A data fusion processing method of pesticide residue detection based on a database is proposed in this study to solve the above-mentioned problems. The method is based on PIDB, APDB, HGDB, and MRLDB on the original data format specification, name specification, information supplementation, and residual level determination. Standardized and complete data stored in the DRDB are formed, as shown in the data fusion processing module in Figure 1. Among them, the residue level determination basis is shown in Table 2, with not detected and level 1 and 2 residues as qualified and level 3 residues as unqualified.

### 2.4. Statistical Analysis and Association Rule Mining

Intelligent analysis methods of statistical analysis and association rule mining are used to analyze the distribution characteristics of the pesticide residue data and discover the implicit association between them.

#### 2.4.1. Statistics and Comparative Analysis

The system mainly helps users analyze the distribution characteristics, exceedance and ranking results of pesticide residues from three different aspects, such as sampling areas, agricultural products and pesticides, by counting, accounting for, ranking, and comparing statistics. The main statistical indicators and calculation methods in this study are shown in Table 3.

#### 2.4.2. Association Rule Mining

Association rule mining is used to mine a frequent pattern P (e.g., item set, subsequence, or substructure) in dataset D and then identify some strong rules from these mined frequent patterns. These strong rules reflect some pattern characteristics and rules hidden in the dataset. The general expression of strong rules is shown in Equation (11).

(11) X⇒Y, in which X⊂P,Y⊂P,X∩Y=ϕ,X∪Y=P

Association rule mining finds frequent item sets by calculating the support degree and then calculates the confidence degree to judge whether strong rules exist in frequent item sets. The support degree refers to the frequency of a pattern P in the dataset. Its calculation method is given as Equation (12).

(12) $$support\left(P\right)=\frac{Number\;of\;data\;items\;containning\;X}{Total\;number\;of\;data\;items\;in\;D}$$

The confidence degree refers to the ratio of the number of data items containing X and Y to the number of data items containing X in the dataset, as shown in Equation (13).

(13) $$confidence\left(X\Rightarrow Y\right)=\frac{Number\;of\;data\;items\;containning\;both\;X\;and\;Y}{Number\;of\;data\;items\;containning\;X\;}$$

Strong rules refer to those rules whose support and confidence are both greater than a given minimum threshold. In this study, we use the Apriori algorithm [33], which is a classic algorithm in association rule mining, to discover the implicit associations in pesticide residue data. The Apriori algorithm is considered the pioneer algorithm for association rule mining. It uses an iterative method called layer-by-layer search to find frequent item sets and mine the strong rules hidden in these frequent item sets [34]. It scans the dataset, accumulates the number of occurrences of each data item, and collects items that meet the minimum support degree. Thus, it obtains a set of frequent 1 item sets, uses frequent 1 item sets to find frequent 2 item sets, uses frequent 2 items to find frequent 3 item sets, and so on until no more frequent k item sets can be found. Then, from the frequent item sets found, strong association rules that meet the minimum confidence are determined. Through these strong association rules, users can quickly obtain the association relationship between pesticide residue data, discover potential risks in pesticide use, and form early warning rules and store them in the PWRDB.

### 2.5. Automatic Report Generation

**Figure 3:**
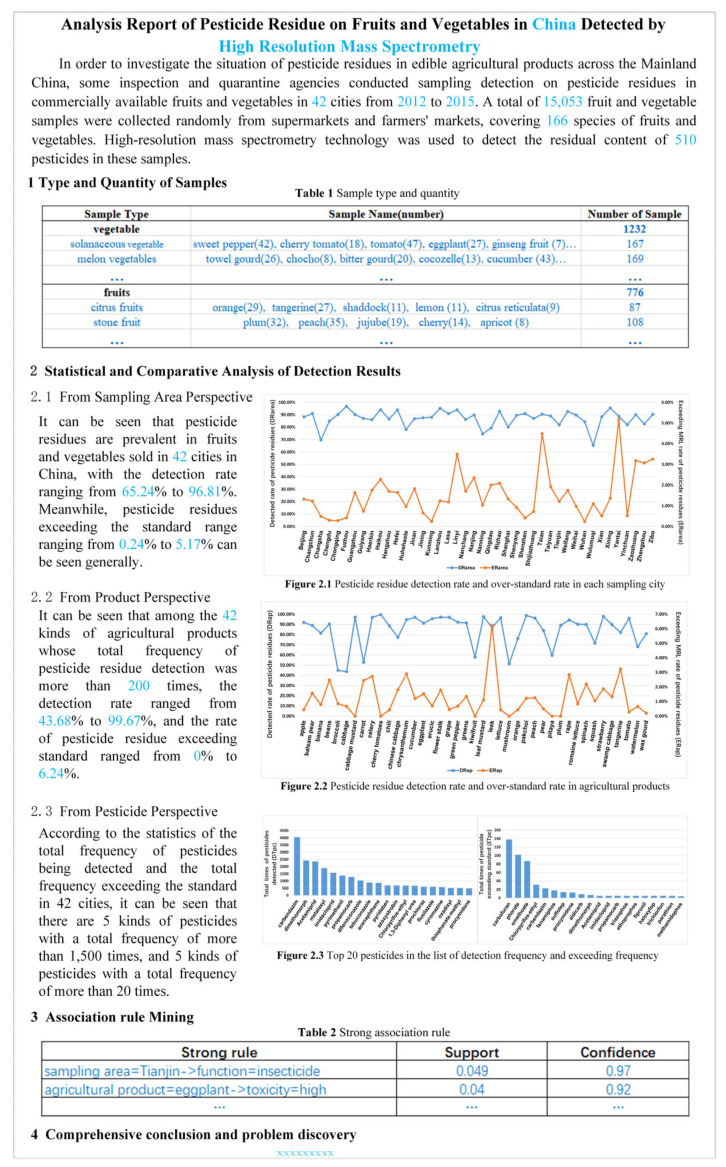
Template of analysis report of pesticide residue detection results.

Manual preparation of the result analysis report requires MRL criteria search, data statistics and analysis, table editing and image insertion, format normalization and layout, and continuous reading and revision of the report content to produce a complete analysis report. This method is tedious, time-consuming, inefficient, and error-prone, and it does not meet the user’s requirements in terms of report accuracy. PRIAS can automatically generate an analysis report of pesticide residue detection results, and thus, it can avoid the problems that can occur in the manual preparation of the report. Sample example is shown in Figure 3, which consists of four parts: (1) statistical analysis of sample types, quantities, and sources; (2) statistical and comparative analysis of pesticide residue data; (3) association rule mining; (4) comprehensive conclusions and problem identification. The report content is divided into two categories: one is the report framework, the title of the table, the style of graphics, and other fixed content, such as the black part in Figure 3. The content of the other blue part is obtained by the intelligent analysis method reading and calculation. The system provides an automatic report generation function by customizing the report template. The generic content is first written into the template and then into the variable content through self-defined data query methods for data reading, intelligent analysis, and chart editing. The specified location is filled to automatically generate the final analysis report of pesticide residue. Each detection agency can export the generated report files through the I/O module to obtain support for their decision making.

## 3. Application Case

We conducted a case study using pesticide residue detection data from 2012 to 2015 in mainland China to illustrate the utility and effectiveness of PRIAS. The detection data included pesticide residue in commercial fruits and vegetables in 42 cities (including 4 municipalities directly under the central government, 27 provincial capitals, and 11 cities with major fruit and vegetable production areas). A total of 15,053 fruit and vegetable samples covering 166 fruit and vegetable species were randomly collected from supermarkets and farmers’ markets. High-resolution mass spectrometry [35] was used to detect the residues of 510 pesticides in these samples. PRIAS was used to process the results as follows.

(1) Data fusion and preprocess

PRIAS performs error checking, information supplementation, and residue level determination on the raw data and then stores them into the DRDB for unified management. A total of 43,851 records were accumulated in DRDB. Each record mainly included attributes such as sample ID, agricultural product name, pesticide name, sampling area, sampling time, the content of pesticide residue, MRL standard value, residue grade, and so on, as listed in Table 4.

(2) Statistical analysis

Statistical analysis of the detection result data from multiple perspectives using the statistical indicators described in Section 2.4.1 and the calculation methods. Additional details can be found in Section 4.1, Section 4.2, Section 4.3.

(3) Association rule mining

Association mining is performed using the Apriori algorithm for selected factors according to the method described in Section 2.4.2. Additional details can be found in Section 4.4.

(4) Automatic generation of result analysis report

PRIAS supports on-demand customization of the analysis report content and “one-click download.” The user can select the data range, such as time range, area range, and so on. The user can also select the statistical functions, such as statistics from a regional perspective, statistics from agricultural products, and statistics from pesticides. A typical result analysis report of roughly 50 pages (including text, figures, and tables) can be generated in about 50 s, which greatly improves the efficiency and accuracy of the report.

## 4. Result and Discussion

### 4.1. Statistics from the Perspective of Sampling Area

**Figure 4:**
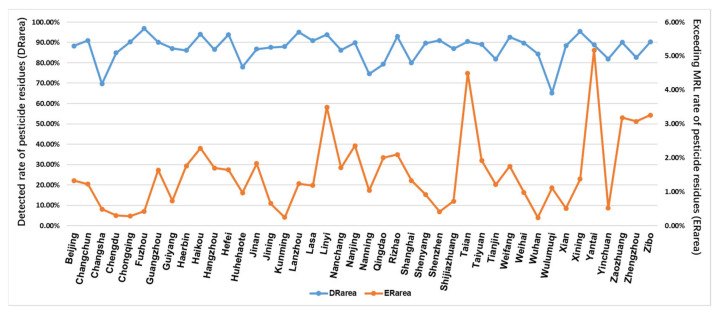
Detected rate of pesticide residues ($DR_{area}$) and exceeding MRL rate of pesticide residue ($ER_{area}$) in each sampling city.

Equations (1) and (2) were used to calculate the detection rate ($DR_{area}$) and exceeding MRL rate ($ER_{area}$) of pesticide residues for each sampling city in China, respectively, and the results are shown in Figure 4. The figure shows that pesticide residues are prevalent in commercially available fruits and vegetables in China and pesticide residues are present in every city. The detection rates range from 65.24% to 96.81%, with most of them above 80%. Further analysis of the pesticide residue exceedance rate shows that pesticide residue exceedance is prevalent, but the exceedance rate is low, which ranges from 0.24% to 5.17%.

### 4.2. Statistics from the Perspective of Agricultural Products

**Figure 5:**
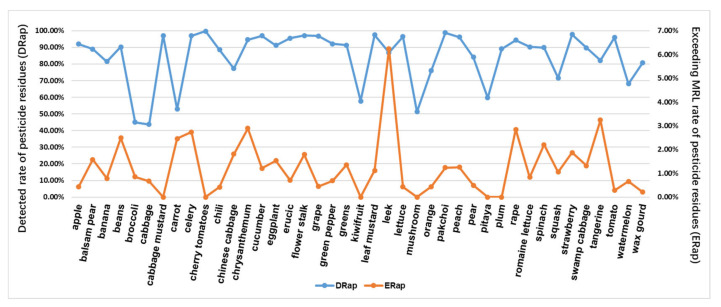
Detected rate of pesticide residues ($DR_{ap}$) and exceeding MRL rate of pesticide residue ($ER_{ap}$) in each agricultural product.

We selected 42 agricultural products with high market sales to ensure sufficient sample size and the types of pesticides tested, and the combined frequency of testing for various pesticide residues in these products exceeded 200 times. The detection rate ($DR_{ap}$) and exceeding MRL rate ($ER_{ap}$) of pesticide residues in each agricultural product were calculated using Equations (5) and (6), respectively, and the results are shown in Figure 5. Pesticide residues are detected in all of the agricultural products that people frequently purchase for use, with detection rates ($DR_{ap}$) ranging from 43.68% to 99.67%, most of which are above 80%, and these findings are of concern. In addition, the analysis of the exceeding MRL rate of pesticide detection in these agricultural products shows that the exceeding MRL rate ($ER_{ap}$) ranges from 0% to 6.24%, with two of them above 3%, which is within a manageable range.

### 4.3. Statistics from the Perspective of Pesticides

**Figure 6:**
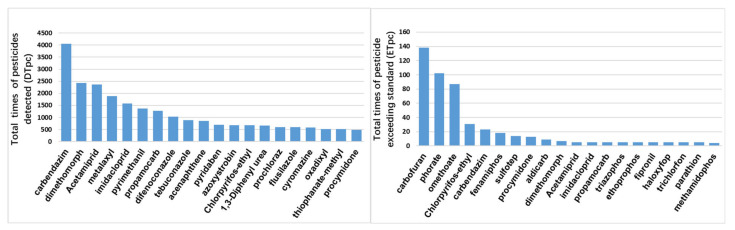
Top 20 pesticides in times detected ($DT_{pc}$) and times of exceeding MRL ($ET_{pc}$).

The number of each pesticide ($DT_{pc}$) detected and the total number of pesticides exceeding MRL ($ET_{pc}$) in 42 cities in China from 2012 to 2015 were calculated using Equations (7) and (8), respectively. The results are shown in Figure 6. Five pesticides have been detected more than 1500 times, namely, carbendazim, dimethomorph, acetamiprid, metalaxyl, and imidacloprid, which indicates that these pesticides are frequently used. In the 4 years, the number of detections exceed 20 times for five pesticides: carbofuran, phorate, omethoate, chlorpyrifos-ethyl, and carbendazim, which need to be monitored.

**Figure 7:**
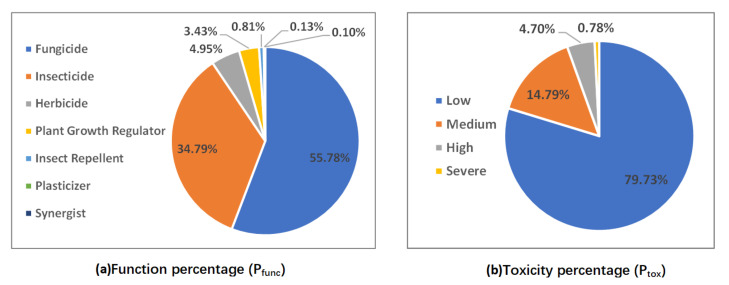
Function percentage ($P_{func}$) and toxicity level percentage ($P_{tox}$) in detected pesticides.

Equations (9) and (10) were used to calculate the percentage of pesticides detected belonging to each function ($P_{func}$) and the percentage of pesticides detected belonging to each toxicity level ($P_{tox}$). The results are shown in Figure 7. Figure 7a reveals that fungicides, insecticides, and herbicides account for more than 90% of the pesticides detected. This result indicates that China still relies on pesticides during this time period to reduce the impact of pests and weeds on crop growth for increasing the yield of agricultural products and ensuring food supply. Figure 7b indicates that, although pesticide residues were widespread in fruits and vegetables sold in China during this time period, most of the pesticide residues are of low to medium toxicity.

### 4.4. Association Rule Mining

In this case, the Apriori algorithm was used to mine the hidden relationships among six factors: sampling area, agricultural product, detected pesticide, the chemical composition of detected pesticide, the toxicity level of detected pesticide, and function of detected pesticide. We selected all records with pesticide residue level 3 (compared with the Chinese MRL standard) from the DRDB, that is, 576 records with pesticide residue exceeding the MRL standard in the 4 years from 2012 to 2015, as the data to be mined. Table 5 lists part of data. The data distribution involved in each mining factor is relatively sparse. Thus, the support of item sets formed by these factors is generally very low. After many experiments, we set the default value of the minimum support in the Apriori algorithm to 0.03 and the default value of the minimum confidence to 0.7.

Table 6 shows the top 5 interesting strong rules mined under the above-mentioned threshold. These strong rules show the hidden associations among the six factors in Table 5. For example, the meaning of the first strong rule is that the highly toxic pesticides detected in Zhengzhou can be 100% inferred to belong to insecticide. This strong rule can guide relevant departments to supervise the use of insecticides in Zhengzhou. The third strong rule means that the highly toxic pesticides detected in celery can be 100% inferred as insecticides, which implies that the use of insecticides in celery needs to be supervised. The fifth strong rule means that for carrots with excessive residues, a 95% chance that the sampling site will be Guangzhou exists, and the pesticide with excessive residues is phorate. This strong rule can remind relevant departments to strengthen the detection and supervision of the pesticide phorate in carrots sold in Guangzhou. Association rule mining can help relevant departments quickly build the association relationship among these six different factors, obtain the early warning rules, and store them in the early warning rule database. Thus, it provides a basis for supervision and decision making.

### 4.5. Discussion

We applied PRIAS to analyze pesticide residue sampling data of commercially available fruits and vegetables in 42 cities in mainland China from 2012 to 2015, from which we discovered the distribution characteristics and potential risks of pesticide residue data and automatically generated pesticide residue sampling analysis reports to improve the accuracy and speed of analysis reports. The application results show that PRIAS can provide decision support for pesticide residue supervision and pre-warning from three aspects: data accumulation, distribution characteristics and potential risk discovery, and automatic generation of analysis reports.

(1) Aggregation and accumulation of pesticide residue detection data

PRIAS continuously collects, fuses, and processes the detection results from different areas and stores them into DRDB, and thus, it realizes the aggregation and accumulation of detection result data. By the end of 2019, DRDB has accumulated the detection results of 8 years since 2012. The MRLDB contains three versions of MRL standards: GB2763-2014, GB2763-2016, and GB2763-2019. The APDB contains 324 kinds of agricultural products and their classification information. The PIDB contains 1100 kinds of pesticide information. All these data provide a reliable and rich database for food safety analysis and supervision.

(2) Pesticide residue distribution characteristics and potential risk discovery

The statistical analysis function provided by the PRIAS helps users analyze pesticide distribution characteristics from multiple perspectives such as sampling areas, agricultural products, and pesticides through detection rate, exceeding MRL rate, and other indicators. Users can then identify the regions, agricultural products, and pesticides that need to be monitored. The association rule mining function provided by the model helps users discover the hidden associations in the data, and the pre-warning information mined through association rules provides a reference for pesticide residue supervision and risk warning.

(3) Automatic generation of sampling analysis reports

Compared with manual analysis, the intelligent analysis function provided by PRIAS greatly improves the accuracy and efficiency of comprehensive analysis. The automatic generation function of the analysis report proposed in this study completes a 50-page comprehensive analysis report on average in about 50 s, including text, data, tables and figures, which provides strong support for timely grasp and reporting of pesticide residue supervision status and pre-warning information.

The limitation of this system currently lies in the fact that the uncertainty of the detection results may be caused by the absence of detection instruments. In the future, we will consider introducing neutrosophic statistical methods [36,37] to solve the data analysis tasks obtained from complex processes or uncertain environments for making the analysis results more adequate and valid.

## 5. Conclusions

In this study, we propose an intelligent analysis system for pesticide residue detection data, namely, PRIAS, which enables online collection, fusion processing, storage, and intelligent analysis of pesticide residue detection data. It supports comprehensive analysis from multiple perspectives, such as agricultural products, pesticides, and sampling areas, to explore the implicit associations in the data and automatically generate analysis reports. The system was applied to analyze the pesticide residue detection data in mainland China from 2012 to 2015. The application results show that PRIAS can greatly improve the depth, accuracy, and efficiency of data analysis and provide support for food safety supervision and decision making. The method can also be easily extended to other data analyses in the food field, such as statistical and risk analysis of sampling data for hazards in other foods.

## Figures and Tables

**Table 1:** Main properties of six databases.

Detection Result Database	MRL Standard Database	Pesticide Info Database	Classified Agri-Product Database	Hierarchical Geographic Database	Pre-Warning Rule Database
Sampling time	Pesticide name	Pesticide name	Sample name	Sampling point	Antecedent of the rule
Sampling point	Agri-product or category	CAS ID	Primary level category	Geographical region	Subsequent of the rule
Sample name	MRL value	Composition	Secondary level category	Provincial level	Support
Pesticide name	Effective time	Function	Tertiary level category	Prefecture level	Confidence
Content of residue	Expiration time	Toxicity		County level	Create time

**Table 2:** Rule of residue level determination.

	Residue Level	Condition
Qualified	Not detected	c ^1^ = 0
	Level 1	0 ≤ c ≤ 0.1 × MRL
	Level 2	0.1 × MRL ≤ c ≤ 1 × MRL
Unqualified	Level 3	c ≥ 1 × MRL

^1^ c represents the content of pesticide residue.

**Table 3:** Statistical indicators and calculation methods.

Aspect	Statistical Indicators	Calculation Methods	Variable Description
Sampling area	(I) $DR_{area}$, the pesticide residue detection rate in each sampling area; (II) $ER_{area}$, the pesticide residue exceeding the MRL rate in each sampling area.	$DR_{area\_i}=\frac{\sum_{j=1}^{m_{i}}d_{ij}}{m_{i}}$ (1) $ER_{area\_i}=\frac{\sum_{j=1}^{m_{i}}e_{ij}}{m_{i}}\;$ (2) $d_{ij}=\left\{\begin{array}{c} 0\;,c_{ij}=0\\ 1\;,\;c_{ij}>0\; \end{array}\right.$ (3) $e_{ij}=\left\{\begin{array}{c} 0,c_{ij}\leq MRL\\ 1,c_{ij}>MRL \end{array}\right.$ (4)	$DR_{area\_i}\;$is the $DR_{area}$ in the ith sampling area; $ER_{area\_i}\;$is the $ER_{area}$ in the ith sampling area; $c_{ij}\;$ is the jth pesticide content detection value in the ith sampling area; $m_{i\;}$ is the total detection frequency of the ith sampling area; i = 1,2,3, …, M (Total of M areas were sampled).
Agricultural Products	(III)$\;DR_{ap}$, the pesticide residues detection rate in various agricultural products; (IV)$\;ER_{ap}$, the pesticide residue exceeding the MRL rate in various agricultural products.	$DR_{ap\_i}=\frac{\sum_{j=1}^{n_{i}}d_{ij}}{n_{i}}$ (5) $ER_{ap\_i}=\frac{\sum_{j=1}^{n_{i}}e_{ij}}{n_{i}}$ (6)	$DR_{ap\_i}$ is the $DR_{ap}\;$ of the ith agricultural product. $ER_{ap\_i}$ is the $ER_{ap}\;$ of the ith agricultural product. $d_{ij}$ and $e_{ij}$ are obtained from Equations (3) and (4), respectively, where $c_{ij}$ is the jth pesticide content detection value of the ith agricultural product. $n_{i}$ is the total detection frequency of the ith agricultural product. i = 1,2,3, …, N (the total of N agricultural products detected).
Pesticides	(V) $DT_{pc}$, the total frequency of various pesticides detected; (VI) $ET_{pc}$, the total frequency of exceeding MRL.	$DT_{pc\_i}=\overset{x_{i}}{\underset{j=1}{\sum }}d_{ij}$ (7) $ET_{pc\_i}=\overset{x_{i}}{\underset{j=1}{\sum }}e_{ij}$ (8)	$DT_{pc\_i}$ is the $DT_{pc}\;$ of the ith pesticide. $ET_{pc\_i}$ is the $ET_{pc}\;$ of the ith pesticide. $d_{ij}$ and $e_{ij}$ are obtained from Equations (3) and (4), respectively, where $c_{ij}$ is the pesticide content detection value of the ith pesticide at the jth time. i = 1,2,3, …, X (Total of X times were detected).
	(VII) $\;P_{func}$, percentage of pesticides detected belonging to each function; (VIII) $P_{tox}$, percentage of pesticides detected belonging to each toxicity level.	$P_{func\_i}=\frac{s_{i}}{y}\times 100\%\;$ (9) $P_{tox\_j}=\frac{t_{j}}{y}\times 100\%$ (10)	$P_{func\_i}$ is the $\;P_{func}\;$ of the ith function. $P_{tox\_j}\;$ is the $P_{tox}\;$ of the jth toxicity level. $s_{i}$ is the number of pesticide species belonging to the ith function, i = 1,2,3, ..., S (S functions of the detected pesticides are considered). $t_{j}$ is the number of pesticide species belonging to the jth toxicity level, j = 1,2,3, ..., T (T toxicity levels of the pesticides detected are considered). y is the number of pesticide species.

**Table 4:** Data records (partially) in DRDB.

Sampling Time	Agricultural Product Name	Sampling Area	Pesticide Name	…	Content of Residue (µg/kg)	MRL (µg/kg)
2015-03-08	apple	Tianjin	etofenprox	…	0.0052	0.6
2014-03-11	leek	Xining	terbufos	…	0.0023	0.01
2013-08-06	potato	Shenyang	pharate	…	0.0013	0.01
2012-07-30	cucumber	Beijing	metalaxyl	…	0.001	0.5
2012-07-30	apple	Beijing	pyrimethanil	…	0.001	7

**Table 5:** Records (partially) for association rule mining.

No.	Sampling Area	Agricultural Product	Pesticide	Chemical Composition	Toxicity Level	Function
1	Haerbing	celery	nitrofen	organochlorine	low	Herbicide
2	Changsha	carrot	phorate	organophosphorus	severe	Insecticide
3	Changsha	celery	carbofuran	carbamates	high	Insecticide
4	Beijing	strawberry	dimethomorph	organic nitrogen	low	Fungicide
5	Beijing	leek	carbendazim	organic nitrogen	low	Fungicide
6	Hefei	romaine lettuce	daminozide	other	low	Plant growth regulator

**Table 6:** First 5 interesting strong association rules.

No.	Rule	Support	Confidence
1	Sampling area = Zhengzhou + toxicity = high ⟹ function = insecticide	0.06	1.0
2	Chemical component = Carbamates + agricultural product = beens ⟹ function = insecticide + toxicity = high	0.06	1.0
3	Toxicity = severe + agricultural product = celery ⟹ function = insecticide	0.049	1.0
4	Toxicity = severe + agricultural product = leek ⟹ function = insecticide	0.042	1.0
5	Agricultural product = carrot ⟹ pestcide = phorate + sampling area = Guangzhou	0.035	0.95

## Data Availability

Data sharing is inapplicable for this article.
